# Podocytes contribute, and respond, to the inflammatory environment in lupus nephritis

**DOI:** 10.1152/ajprenal.00512.2017

**Published:** 2018-09-12

**Authors:** Rachael D. Wright, Michael W. Beresford

**Affiliations:** ^1^Department of Women’s and Children’s Health, Institute of Translational Medicine, University of Liverpool, Liverpool, UK; ^2^Department of Paediatric Rheumatology, Alder Hey Children’s NHS Foundation Trust, Liverpool, UK

**Keywords:** biomarkers, effacement, inflammation, lupus nephritis, podocytes

## Abstract

Lupus nephritis (LN) affects up to 80% of juvenile onset systemic lupus erythematosus patients, leading to end stage renal failure requiring dialysis or transplantation in 10–15%. Podocytes are specialized epithelial cells of the glomerulus known to be a key site of damage in glomerular diseases. However, their roles in LN have yet to be fully identified. This project aims to identify structural and functional roles of podocytes in an in vitro model of LN. Conditionally immortalized podocytes were treated with proinflammatory cytokines (IL-1β, TNF-α, IFN-α, and IFN-γ) alone and in combination in an in vitro model of LN and were assessed for their structural and functional characteristics. Podocytes produce TNF-α, IL-6, IL-8, VEGF, granulocyte-monocyte colony stimulating factor (GM-CSF), and macrophage colony stimulating factor (M-CSF) at relatively low levels under basal conditions; stimulation with IL-1β led to increased secretion of IL-6 (*P* = 0.011), IL-8 (*P* = 0.05), VEGF (*P* = 0.02), and M-CSF (*P* = 0.03). Stimulation with TNF-α led to increased secretion of M-CSF (*P* = 0.049) and stimulation with IFN-γ led to novel production of IL-10 (*P* = 0.036) and interferon-γ-inducible protein-10 (*P* = 0.036). Podocytes demonstrate a reduction in the area covered by filamentous-actin in response to IL-1β treatment within 1 h (*P* = 0.011), which is restored by 24 h, associated with an increase in the level of intracellular calcium but not with increased cell death. Podocytes contribute to the inflammatory milieu in LN through cytokine/chemokine secretion and respond to the inflammatory milieu via rearrangement of the actin cytoskeleton leading to effacement, a well-known method of protection against apoptosis in these cells. This demonstrates that podocytes are involved in the pathogenesis of LN.

## INTRODUCTION

Lupus nephritis (LN) is one of the most common and damaging manifestations of juvenile-onset systemic lupus erythematosus (JSLE), affecting up to 80% of patients. LN can lead to end stage renal failure in ~10–15% of patients (17, 18, 26, 40). LN is a remitting and relapsing clinical feature of JSLE, and each flare increases the risk of permanent kidney damage (3). LN is initiated by the binding of autoantibodies to antigens expressed by native kidney cells (29) leading to an inflammatory response and organ damage (3).

The current gold standard for diagnosing a flare of LN is renal biopsy. However, this is invasive and in children requires a general anesthetic; thus, it is not suitable for repetition (35). A recent study has also challenged the interobserver reliability of biopsy assessment (30). Studies in our laboratories have identified a panel of biomarkers detectable in the urine of patients during an LN flare that correlates with disease activity with high sensitivity and specificity (36, 39, 41, 42). These include α-1-acid glycoprotein (AGP), caeruloplasmin (CP), transferrin (Tf), lipocalin-type prostaglandin D2 synthase (L-PGDS), monocyte chemoattractant protein-1 (MCP-1), VCAM-1, and neutrophil gelatinase-associated lipocalin (NGAL). These urinary biomarkers are either produced systemically and filtered into the urine due to damage/loss of the glomerular filtration barrier, or produced locally by native kidney cells/infiltrating immune cells in response to damage.

Podocytes are specialized epithelial cells of the glomerulus that are highly involved in selective filtration by providing a charge and size-specific barrier to proteins and blood cells (31). Neighboring podocytes have interdigitating foot processes with slit diaphragms connecting them, that wrap around the glomerular capillary, forming a barrier and preventing the migration of proteins and solutes from the circulation into the urine (12). Podocyte damage is a key step in the progression of glomerular disease; many hereditary renal diseases are caused by mutations in genes that encode podocyte proteins (such as Alport syndrome and congenital nephrotic syndrome of the Finnish type) (6). Furthermore, podocyte depletion in rats using diphtheria toxin led to glomerulosclerotic disease, supporting the role of these cells in its pathogenesis (43). Few studies to date have assessed the role of podocytes in the pathogenesis of LN.

This study aimed to assess the role of podocytes in LN first by investigating their role in the production of known urinary biomarkers and then by assessing both their contribution, and their response to, the inflammatory environment seen in LN, using an in vitro cytokine-induced model of disease.

## MATERIALS AND METHODS

### 

#### Materials.

All recombinant cytokines were purchased from Peprotech, London, UK.

#### Human podocyte culture.

Human podocytes were provided by Moin Saleem (Children’s Renal Unit and Academic Renal Unit, University of Bristol, Southmead Hospital, Bristol, UK). These cells were conditionally immortalized using the temperature-sensitive large T antigen-SV-40 transgene as previously described (33). These cells have been shown to differentiate fully by 10 to 14 days after switching from 33°C to 37°C. Cell passages between 15 and 30 were used in all experiments. Podocytes were routinely cultured in RPMI-1640 medium with L-glutamine (Lonza, Leeds, UK) supplemented with 10% fetal calf serum (ThermoScientific) and insulin transferrin selenium (Sigma-Aldrich, Dorset, UK).

After 10 to 12 days of differentiation, podocytes were treated with cytokines designed to model the inflammatory environment of the kidney in LN patients; IL-1β, TNF-α, IFN-α and IFN-γ (all known to be involved in the pathogenesis of LN) at 10 ng/ml each, alone and in combination. Following 24-h incubation, conditioned media was collected from each well and stored at −80°C for ELISA and multiplex analyses, and RNA was extracted using TRIzol (ThermoScientific).

#### qRT-PCR.

RNA was extracted from podocytes using the RNeasy miniprep kit (Qiagen, Manchester, UK), following the manufacturer’s instructions. The RNA concentration was determined by Nanodrop (Nanodrop 1000, ThermoScientific) at 260 nm, purity was assessed using the 260/280 nm ratio, and 200 ng RNA was transcribed into cDNA using the AffinityScript multitemp cDNA synthesis kit (Agilent Technologies, Cheshire, UK) following the manufacturer’s instructions. qRT-PCR was performed using the primers described in (Table 1) with the Brilliant III Ultra-fast SYBR QPCR master mix kit (Agilent Technologies) following the manufacturer’s instructions. The geometric mean of tyrosine 3-monooxygenase/tryptophan 5-monooxygenase activation protein Zeta (YWHAZ), TATA-box-binding protein (TBP), and glyceraldehyde 3-phosphate dehydrogenase (GAPDH) was used as an internal reference control for normalization.

**Table 1.** List of primers used for qRT-PCR

Gene	Forward Primer	Reverse Primer
*YWHAZ*	ACTGGGTCTGGCCCTTAACT	GGGTATCCGATGTCCACAATGTC
*TBP*	GTGACCCAGCATCACTGTTTC	GAGCATCTCCAGCACACTCT
*GAPDH*	ATGGCTATGATGGAGGTCCAG	TTGTCCTGCATCTGCTTCAGC
*MCP-1*	CTCGCTCAGCCAGATGCAAT	TCTCCTTGGCCACAATGGTC
*CP*	ACAGCACCTGGAAGTGACTC	AGGGCCTCTCTCCTTTCGAT
*NGAL*	ACCCTCTACGGGAGAACCAA	AGCTCCCTCAATGGTGTTCG
*VCAM-1*	GGTGGGACACAAATAAGGGT	GCTTGAGAAGCTGCAAAC
*L-PGDS*	CCAGGGCTGAGTTAAAGGAGA	CCCTGGGGAGTCCTATTGTTC
*AGP*	GCCAAGAGCATTTCGCTCAC	CTGGCTTGTCAGCATAGACAGA
*Tf*	AACCAGGCCCAGGAACATTT	AGATTCCGGATGGCAGTGAC

YWHAZ, tyrosine 3-monooxygenase/tryptophan 5-monooxygenase activation protein Zeta; TBP, TATA-box-binding protein; MCP-1, monocyte chemoattractant protein-1; CP, caeruloplasmin; NGAL, neutrophil gelatinase-associated lipocalin; L-PGDS, lipocalin-type prostaglandin D2 synthase; AGP, α-1 acid glycoprotein; Tf, transferrin.

#### ELISA.

MCP-1, VCAM-1, and NGAL DuoSets were purchased from R&D Systems, Abingdon, UK, and CP ELISA kits were ordered from Universal Biologicals, Cambridge, UK. All were performed according to the manufacturer’s instructions to assay protein levels in conditioned media from cytokine-treated podocytes.

#### Multiplex.

A Luminex magnetic bead assay was purchased from R&D Systems, Abingdon UK, which was able to detect TNF-α, IL-6, IL-8, interferon-γ-inducible protein (IP)-10, IL-10, VEGF, GM-CSF, and M-CSF. The assay was performed according to manufacturer’s instructions to assay protein levels in conditioned media from cytokine-treated podocytes. The plate was read using a Merck Millipore Luminex MAGPIX analyzer.

#### Actin cytoskeletal reorganization assessment.

Podocytes were seeded onto coverslips in a 12-well plate and following differentiation were treated with cytokines as described above for 1 h and 24 h. Additionally, podocytes were treated with 10% sera from patients with active LN by British Isles Lupus Assessment Group (renal BILAG A/B), inactive LN (renal BILAG D/E) or age- and sex-matched healthy controls for 1 h. These cells were then fixed in 4% formalin in PBS, permeabilized using PBS with 0.1% Triton X-100, and the actin cytoskeleton was stained with Alexa Fluor 488 phalloidin (Thermo Fisher Scientific). The coverslips were mounted using ProLong gold antifade mount with DAPI (Thermo Fisher Scientific). Images were taken on a Leica TCS SPE confocal microscope, and five random images per coverslip were collected in which a minimum of three nuclei were in each field of view. ImageJ software was used to analyze the area of phalloidin stain per field of view. Briefly, a threshold was set on the 488-nm channel, and the software determined the number of pixels above this threshold and the area.

#### Apoptosis assessment.

To determine whether apoptotic cells are being lost into the media, podocytes were seeded in six–well plates. Following differentiation, cells were treated with cytokines for 1 h as previously explained. The conditioned media containing any detached podocytes was collected, and the cells were trypsinized and collected and pooled with the conditioned media. These cells were then assayed for Annexin V and propidium iodide (PI) staining (Annexin V FITC apoptosis detection kit, Sigma-Aldrich) according to manufacturer’s instructions by using a Merck Guava flow cytometer.

#### Calcium mobilization assay.

Calcium mobilization was assessed using the Molecular Probes fluo-4 NW calcium assay kit (Thermo Fisher Scientific) per the manufacturer’s instructions. Equal numbers of podocytes were plated onto a 96-well plate and left to differentiate for 10 days; following this they were washed and resuspended in 50 μl PBS, and 50 μl of the dye-loading solution in calcium-free media was added per well. The plate was incubated at 37°C for 30 min; then cells were stimulated with cytokines, as previously described, for 15 min at 37°C. Kinetic assessment of calcium mobilization was performed using a BMG OMEGA FluoStar microplate reader (excitation at 485 nm, emission at 520 nm) with ionomycin (1 μg/ml, Sigma-Aldrich) administration at 20 s, and readings were taken until 80 s. Data were analyzed using BMG MARS software.

#### Albumin permeability assays.

Equal numbers of podocytes were seeded onto gelatin-coated 3-μm transwell inserts (EMD Millipore, Watford, UK) and allowed to differentiate for 10–12 days. Following differentiation, cells were treated for 1 h with cytokines known to be involved in LN as previously described. The inserts were washed and 1 ml RPMI was added to the bottom of the well, 500 μl RPMI (+2 mg/ml BSA) was loaded to the top of the insert, and the cells were incubated at 37°C for 90 min. The insert was discarded and the culture media in the well was assayed for albumin content using a bicinchoninic acid assay (Thermo Fisher Scientific).

#### Statistical analysis.

Data are expressed as median (and range) unless otherwise stated. Statistical analysis was performed using GraphPad Prism 7.01 software program. Statistical significance was evaluated using Friedman’s test (for paired analyses) or Kruskal-Wallis test with Dunn’s post hoc test. A *P* value < 0.05 was statistically significant.

## RESULTS

### 

#### Podocytes secrete low levels of biomarkers in a model of LN.

**Fig. 1.**
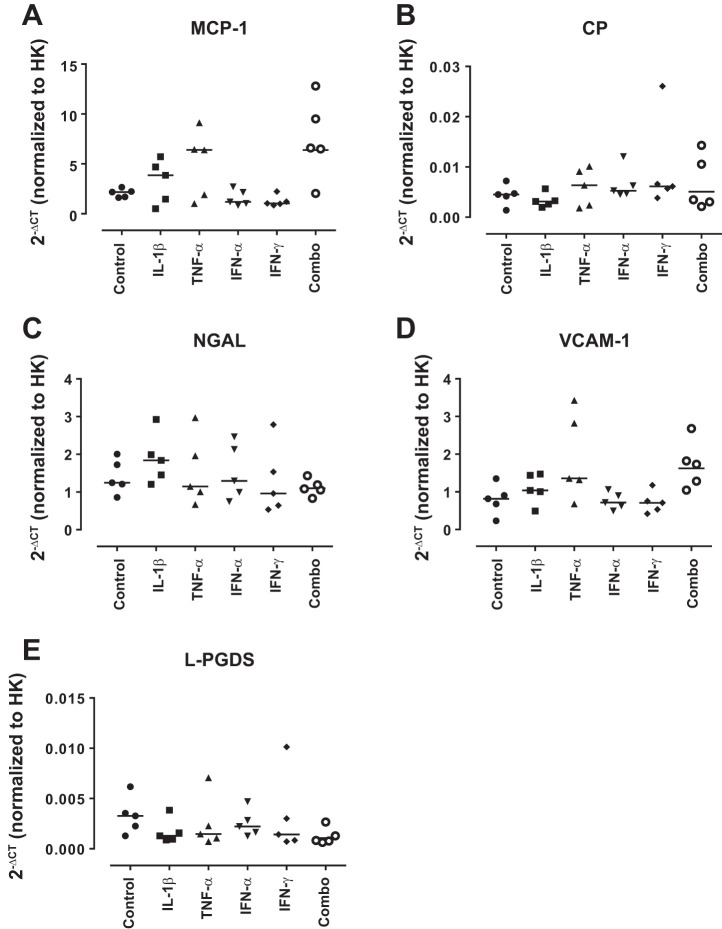
Lupus nephritis biomarker mRNA expression by conditionally immortalized podocytes following cytokine treatment. Conditionally immortalized podocytes were treated with IL-1β, TNF-α, IFN-α, and IFN-γ alone and in combination (Combo). mRNA expression was assessed for monocyte chemoattractant protein-1 (MCP-1; *A*), caeruloplasmin (CP; *B*), neutrophil gelatinase-associated lipocalin (NGAL; *C*), VCAM-1 (*D*), and lipocalin-type prostaglandin D2 synthase (L-PGDS; *E*) normalized to the geometric mean of tyrosine 3-monooxygenase/tryptophan 5-monooxygenase activation protein Zeta (YWHAZ), TATA-box binding protein (TBP), and GAPDH. HK, housekeeping. *N* = 5 per group; data are analyzed using Friedman’s test with Dunn’s post hoc test.

To investigate the role of podocytes in the production of known urinary biomarkers of LN, conditionally immortalized podocytes were treated with cytokines known to be involved in LN (IL-1β, TNF-α, IFN-α, and IFN-γ alone and in combination) for 24 h. Low expression of mRNA for MCP-1, CP, NGAL, L-PGDS, and VCAM-1 were seen in untreated cells; however, these were not modulated following cytokine treatments (Fig. 1, *A*–*E*). AGP and Tf were not expressed by podocytes under any condition (data not shown).

**Fig. 2.**
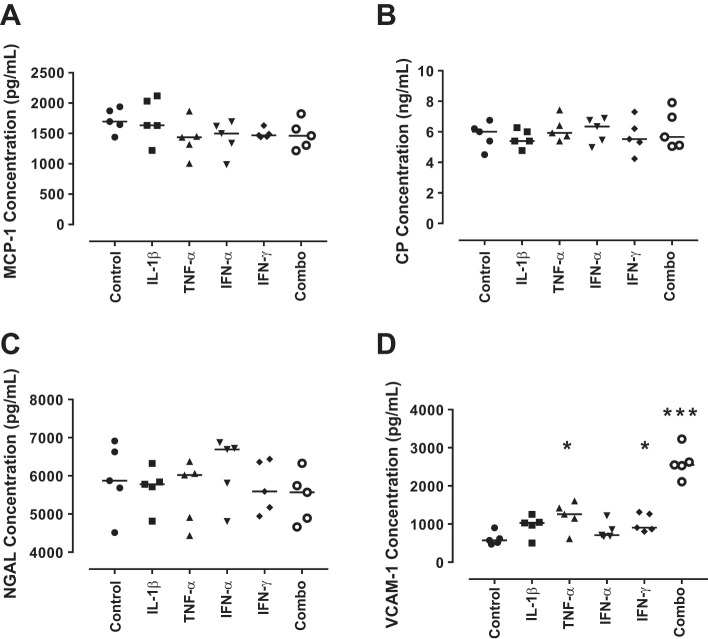
Lupus nephritis biomarker expression by conditionally immortalized podocytes following cytokine treatment. Conditionally immortalized podocytes were treated with IL-1β, TNF-α, IFN-α, and IFN-γ alone and in combination (Combo). ELISA was used to assess protein levels of MCP-1 (*A*), CP (*B*), neutrophil gelatinase-associated lipocalin (NGAL; *C*), and VCAM-1 (*D*). *N* = 5 per group; data were analyzed using Friedman’s test with Dunn’s post hoc test. *P < 0.05 and ***P < 0.001 vs. control.

Following this, the protein expression of the detectable biomarkers was assessed using ELISA. MCP-1, NGAL, and CP were expressed by podocytes at low levels under basal conditions but were not modulated by any of the experimental conditions tested (Fig. 2, *A*–*C*). VCAM-1 was expressed by untreated podocytes (570.5 pg/ml, range 471.9–903.1 pg/ml). This was significantly increased by TNF-α treatment (1,256 pg/ml, range 618.4–1,607 pg/ml; *P* = 0.02), IFN-γ treatment (906.8 pg/ml, range 805.4–1,313 pg/ml; *P* = 0.049) and with the combination of cytokines (2,551 pg/ml, range 2,110–3,229 pg/ml; *P* < 0.0001) (Fig. 2*D*). L-PGDS levels were below the level of detection for the ELISA (data not shown).

These data demonstrated that in response to cytokines known to be involved in the pathogenesis of LN, relatively low levels of urinary biomarker expression were detected within this in vitro model. This suggests that podocytes are not the major source of these urinary proteins in LN.

#### Podocytes contribute to the inflammatory milieu in a model of LN.

**Fig. 3.**
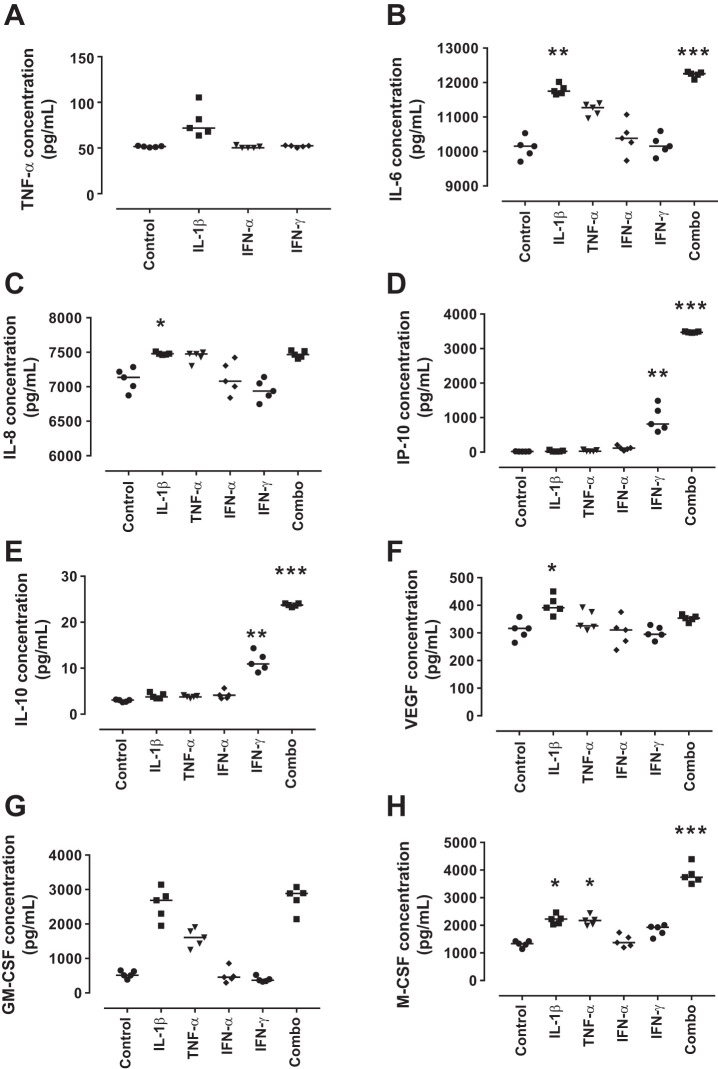
Cytokine/chemokine expression by conditionally immortalized podocytes following cytokine treatment. Conditionally immortalized podocytes were treated with IL-1β, TNF-α, IFN-α, and IFN-γ alone and in combination (Combo). Multiplex was used to assess protein levels of TNF-α (*A*), IL-6 (*B*), IL-8 (*C*), interferon-γ-inducible protein (IP)-10 (*D*), IL-10 (*E*), VEGF (*F*), granulocyte-monocyte colony stimulating factor (GM-CSF; *G*), and macrophage colony stimulating factor (M-CSF; *H*). *N* = 5 per group; data were analyzed using Friedman’s test with Dunn’s post hoc test. *P < 0.05, **P < 0.05, and ***P < 0.001 vs. control.

To determine the contribution of podocyte-derived cytokines to the inflammatory environment, supernatants from the cytokine-treated podocytes were assayed via multiplex analysis for levels of TNF-α, IL-6, IL-8, IP-10, IL-10, VEGF, GM-CSF, and M-CSF (Fig. 3), cytokines previously determined to be secreted from podocytes in response to injurious stimuli (5, 8, 9, 11, 14, 20, 27). Podocytes expressed IL-6 under basal conditions (10,152 pg/ml, range 9,706–10,532 pg/ml), and this was significantly increased following treatment with IL-1β (11,750 pg/ml, range 11,658–12,016 pg/ml; *P* = 0.011) and the combined cytokine treatment (12,257 pg/ml, range 12,088–12,313 pg/ml; *P* = 0.0005) (Fig. 3*B*). IL-8 was expressed at high levels under basal conditions (7,135 pg/ml, range 6,876–7,287 pg/ml), and this was significantly increased by treatment with IL-1β (7,479 pg/ml, range 7,462–7,510 pg/ml; *P* = 0.05) (Fig. 3*C*). IP-10 was expressed at low levels under basal conditions (16.28 pg/ml, range 14.94–24.79 pg/ml); this increased significantly upon treatment with IFN-γ (811.8 pg/ml, range 589.9–1,487 pg/ml; *P* = 0.0036) and with the combined treatment (3,473 pg/ml, range 3,456–3,498 pg/ml; *P* < 0.0001) (Fig. 3*D*). A similar trend to IP-10 was seen with IL-10 levels with relatively low-level expression of IL-10 under basal conditions (3.068 pg/ml, range 2.599–3.186 pg/ml), that significantly increased upon treatment with IFN-γ (10.94 pg/ml, range 9.059–14.35 pg/ml; *P* = 0.0036), and the combined treatment (23.74 pg/ml, range 23.27–24.1 pg/ml; *P* = 0.0001) (Fig. 3*E*). VEGF was expressed at basal levels (316.5 pg/ml, range 264.6–358 pg/ml), and this was significantly increased in response to IL-1β (391.2 pg/ml, range 359.7–450.1 pg/ml; *P* = 0.02) (Fig. 3*F*). Levels of M-CSF were relatively high at basal conditions (1,336 pg/ml, range 1,142–1,424 pg/ml), and this was significantly increased following treatment with IL-1β (2,227 pg/ml, range 2,033–2,465 pg/ml; *P* = 0.03), TNF-α (2,174 pg/ml, range 1,984–2,438 pg/ml; *P* = 0.049), and the combined treatment (3,743 pg/ml, range 3,496–4,395 pg/ml; *P* = 0.0005) (Fig. 3*H*). No differences were seen in levels of TNF-α or GM-CSF under any of the experimental conditions.

These data demonstrate that podocytes secrete some of the cytokines and chemokines responsible for leukocyte recruitment and maturation in response to the inflammatory environment seen in LN, suggesting they may play a role in the perpetuation of this inflammatory response.

#### Inflammatory stimulation of podocytes leads to effacement not associated with apoptosis.

**Fig. 4.**
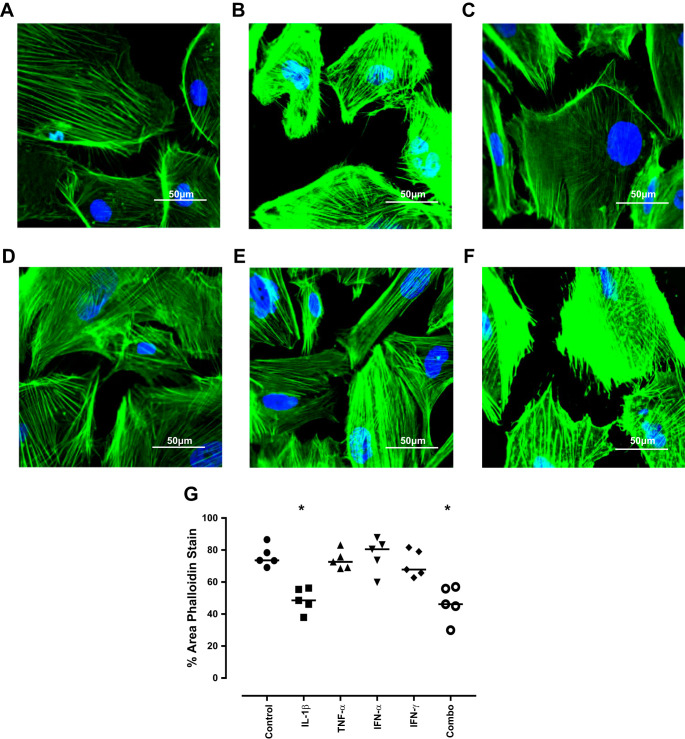
Actin cytoskeletal rearrangement of podocytes following cytokine treatment. Conditionally immortalized podocytes were treated with IL-1β, TNF-α, IFN-α, and IFN-γ alone and in combination (Combo). Phalloidin staining was used to assess reorganization of the podocyte actin cytoskeleton in response to cytokine treatment. Representative images of control (*A*), IL-1β (*B*), TNF-α (*C*), IFN-α (*D*), IFN-γ (*E*), and a combination of all four cytokines (*F*). Graph of median % area values at 1 h (*G*). *N* = 5 per group; data were analyzed using Friedman’s test with Dunn’s post hoc test. *P < 0.05 vs. control.

Podocyte loss is of critical importance to the pathogenesis of various glomerular diseases including diabetic nephropathy, focal segmental glomerulosclerosis, HIV-associated nephropathy and IgA nephropathy, among others (44). A decrease in the number of podocytes and exposure of the underlying glomerular basement membrane is seen in human biopsy tissue and animal models of disease with a concurrent increase in the number of podocytes detected in the urine that correlates with disease activity (15, 16, 28). Thus, confocal microscopy was used to visualize the filamentous (F)-actin cytoskeleton via phalloidin staining following cytokine stimulation of the podocytes. Untreated podocytes exhibited a healthy phenotype with confluent cells covering most of the area imaged (73.55%, range 69.09–86.54%) (Fig. 4*A*). Upon treatment of the podocytes with IL-1β, a decrease in the average area covered by cells was seen (48.54%, range 37.95–56.27%; *P* = 0.011) (Fig. 4*B*). A significant decrease in area was also seen following the combination of cytokine treatments (46.13%, range 29.96–56.99%; *P* = 0.020) (Fig. 4*F*). No other specific cytokine treatment had a statistically significant effect on the area covered by podocytes at 1 h (Fig. 4*G*).

**Fig. 5.**
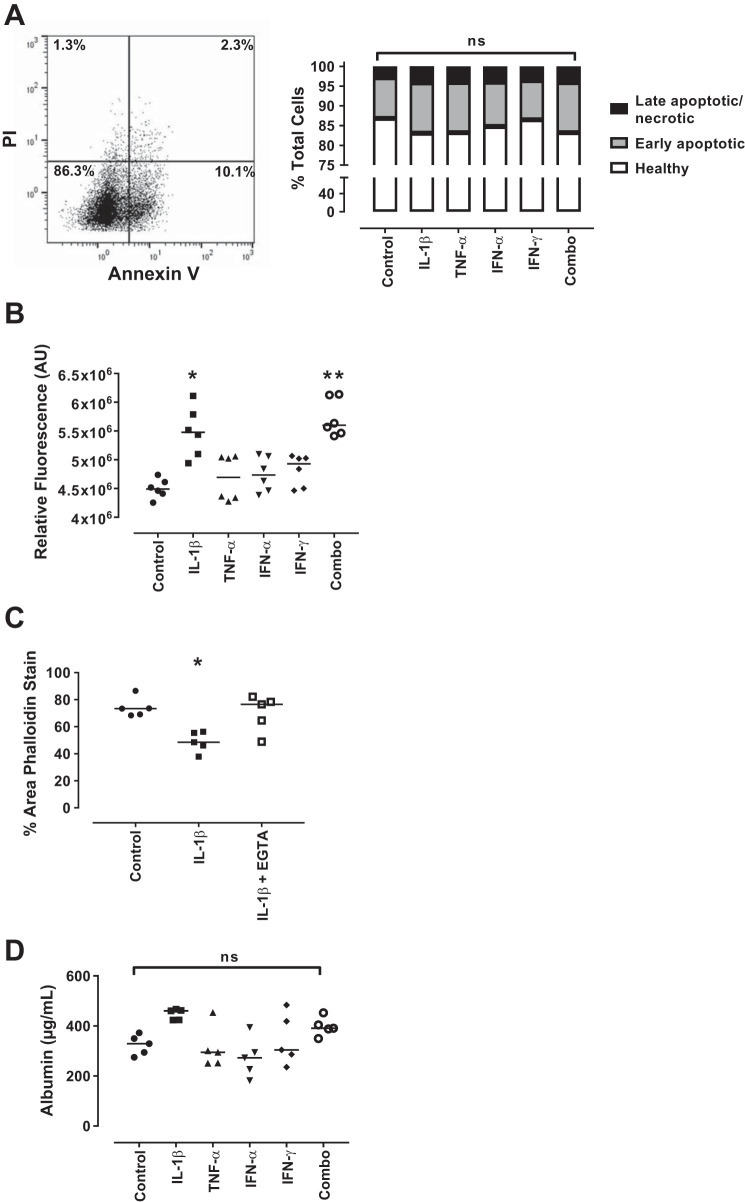
Assessment of podocyte function following cytokine treatment. Conditionally immortalized podocytes were treated with IL-1β, TNF-α, IFN-α, and IFN-γ alone and in combination (Combo). Culture media and trypsinized podocytes were collected following inflammatory stimulation and cell death was assessed using Annexin V/PI binding after 1 h stimulation. Representative dot plot (control) and graph (*A*). Intracellular calcium mobilization was assessed after 15 min stimulation (*B*), phalloidin staining was used to assess reorganization of the podocyte actin cytoskeleton following pretreatment with EGTA (*C*), and the permeability of a podocyte monolayer to albumin was assessed following 1 h stimulation (*D*). PI, propidium iodide; AU, arbitrary units; ns, not significant. *N* = 5 per group; data were analyzed using Friedman’s test with Dunn’s post hoc test. *P < 0.05 and **P < 0.005 vs. control.

To confirm that the reduction in cell area was not due to cell death, podocytes were analyzed for Annexin V/PI expression. As dying podocytes can lose their adherent properties and thus be found in the media, conditioned media was collected alongside trypsinized cells and assayed. We determined that no differences in the number of early apoptotic (Annexin V^+^/PI^−^) or late apoptotic/necrotic cells (Annexin V^+^/PI^+^) could be seen with any of the treatments, confirming the reduction in exposed area seen via phalloidin staining is not due to apoptosis or associated cellular loss (Fig. 5*A*).

An increase in the level of intracellular calcium as determined by calcium mobilization assessment was seen before actin cytoskeletal reorganization. Untreated podocytes exhibited a characteristic mobilization of calcium with an increase in response to the ionophore (ionomycin) that steadily decreased over the 80-s analysis period area under the curve (AUC) = 4.49 × 10^6^, range 4.25 × 10^6^ – 4.74 × 10^6^. This was significantly increased in IL-1β-treated podocytes (AUC = 5.47 × 10^6^, range 4.93 × 10^6^ − 6.10 × 10^6^; *P* = 0.01) and in the cytokine combination treatment (AUC = 5.60 × 10^6^, range 5.41 × 10^6^ – 6.13 × 10^6^; *P* = 0.003) (Fig. 5*B*) but was unaffected by any other treatment.

To confirm that the calcium mobilization is preceding the reorganization of the cytoskeleton, podocytes were treated with IL-1β to induce a decrease in F-actin area (46.72%, range 32.18–58.36%; *P* = 0.03) compared with control (73.42%, range 68.38–89.17%) as expected. Podocytes were then pretreated with 0.3 mM ethylene glycol-bis (β-aminoethyl ether)-N,N,N',N'-tetraacetic acid (EGTA) for 15 min to chelate calcium before addition of cytokines. This rescued the reduction in F-actin area (76.55%, range 48.91–82.18%), confirming that calcium mobilization is required for F-actin reorganization (Fig. 5*C*).

Cytoskeletal reorganization resulted in areas uncovered by podocytes. As this may be contributing to the proteinuria seen in LN, the permeability of the monolayer to albumin was assessed. Untreated podocytes allowed migration of a basal level of albumin (329.4 μg/ml, range 274.9–372.4 μg/ml), and while this appeared higher in IL-1β-treated cells (460 μg/ml, range 423.6–466.9 μg/ml; *P* = 0.06), it failed to reach statistical significance (Fig. 5*D*).

These data demonstrate that in response to the cellular damage caused in our model, there is increased intracellular calcium mobilization within the podocytes leading to a reorganization of the actin cytoskeleton, not associated with an increase in cellular apoptosis.

#### Acute inflammatory stimulation causes a transient reduction in F-actin expression.

**Fig. 6.**
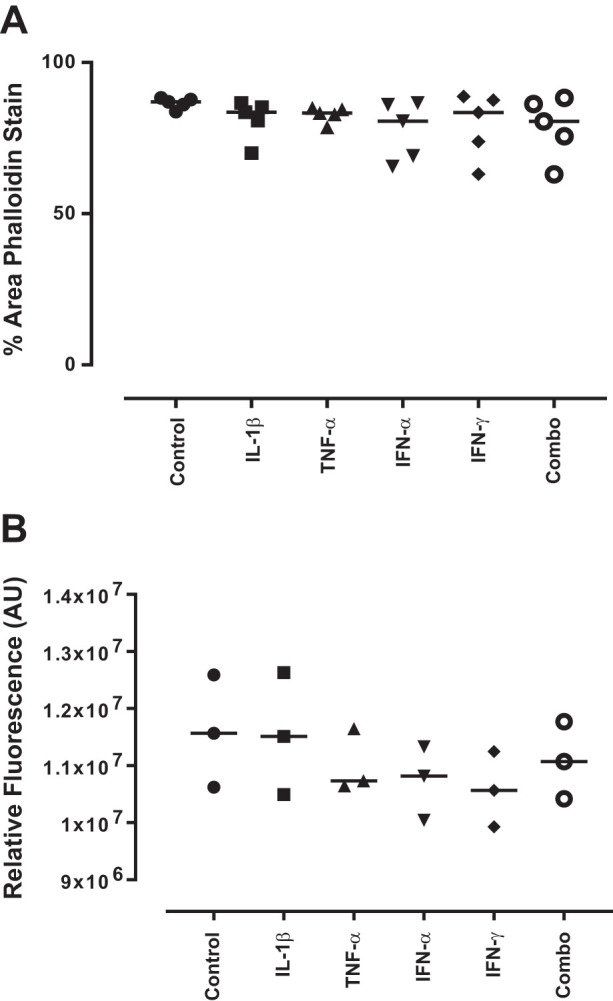
Actin cytoskeletal rearrangement of podocytes following 24-h cytokine treatment. Conditionally immortalized podocytes were treated for 24 h with IL-1β, TNF-α, IFN-α, and IFN-γ alone and in combination (Combo). Phalloidin staining was used to assess reorganization of the podocyte actin cytoskeleton in response to 24-h cytokine treatment (*A*), intracellular calcium mobilization was assessed after 15-min stimulation (*B*). AU, arbitrary units. *N* = 3–5 per group; data were analyzed using Friedman’s test with Dunn’s post hoc test.

The F-actin cytoskeleton of podocytes following inflammatory stimulation was also assessed at 24 h. At this time point, the reduction in area had reversed and levels were similar to those seen at 1 h in untreated cells for all conditions. This indicates that effacement is occurring in these cells following inflammatory stimulation, which is resolved following removal of the stimulus (Fig. 6*A*). Additionally, the calcium mobilization following 24-h stimulation was assessed, and no significant differences were seen between treatments (Fig. 6*B*).

The reduction in cell area was restored by 24 h and no change in calcium mobilization could be detected between treatments, indicating effacement is a temporary process in response to acute damage.

#### Actin reorganization was not induced by LN patient sera.

**Fig. 7.**
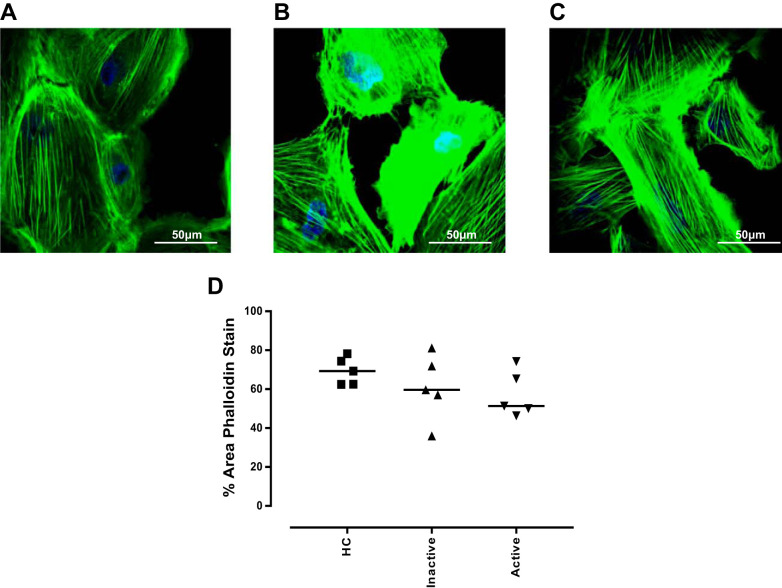
Actin cytoskeletal rearrangement of podocytes following patient sera treatment. Conditionally immortalized podocytes were treated for 1 h with serum from age- and sex- matched healthy controls (*A*), patients with active lupus nephritis (LN; renal BILAG A/B) (*B*), and inactive LN (renal BILAG D/E) (*C*). Phalloidin staining was used to assess reorganization of the podocyte actin cytoskeleton in response to cytokine treatment. Graph of median % area values at 1 h (*D*). HC, healthy controls; BIPAG, British Isles Lupus Assessment Group. *N* = 5 per group; data were analyzed using Friedman’s test with Dunn’s post hoc test.

To investigate a more physiologically relevant model, conditionally immortalized podocytes were incubated for 1 h with 10% sera from patients with active LN (renal BILAG A/B), inactive LN (renal BILAG (D/E), or age- and sex-matched healthy controls (Table 2). Podocytes treated with healthy control sera covered a large proportion of the area imaged (69.28%, range 62.47–78.23%) (Fig. 7*A*), this was not modulated by either inactive (59.66%, range 35.96–81.19%) (Fig. 7*B*) or active (51.31%, range 46.36–74.24%) (Fig. 7*C*) LN sera (Fig. 7*D*).

**Table 2.** Demographics, renal BILAG scores and medication for lupus nephritis patients

Demographics	Active LN	Inactive LN	Healthy Controls
*n*	5	5	5
Age; years, (median range)	16.76 (14.92–18.29)	16.83 (14.89–18.27)	16.74 (14.93–17.46)
Age at diagnosis; years, (median range)	12.78 (10.5–14.52)	15.9 (11.46–16.88)	
Females, (%)	100 (5)	100 (5)	100 (5)
Nationality, (%)			
White British	100 (5)	80 (4)	80 (4)
Chinese	0	20 (1)	0
Other white background	0	0	20 (1)
Renal BILAG domains			
Renal Hypertension, (%)	80 (5)	0	
Urine ACR, mg/dl, (median range)	1.15 (0–7.4)	0.85 (0.7–4.2)	
Renal creatinine, mg/dl, (median range)	55 (49–68)	60 (53–80)	
Estimated GFR, ml · min · 1.73 m^2^, (median range)	101.5 (49.8–118.8)	108.7 (83–124.2)	
Medications, *n*			
Hydroxychloroquine	4	4	
Azathioprine	0	1	
Mycophenolate mofetil	2	2	
Prednisolone	3	1	
Methotrexate, oral	1	0	
Rituximab	2	0	
Cyclophosphamide	1	1	

LN, lupus nephritis; ACR, albumin creatinine ratio; GFR, glomerular filtration rate; BIPAG, British Isles Lupus Assessment Group.

## DISCUSSION

JSLE is a complex, systemic autoimmune disease that severely affects the kidney more than the adult-onset disease does (34). Each renal flare of LN increases the accrual of end organ damage until end stage renal disease occurs. Podocyte loss is of critical importance to the pathogenesis of other glomerulosclerotic diseases. The terminally differentiated nature of these cells means a loss of >20% causes progressive sclerosis (43), and thus the role of podocytes in juvenile-onset LN was investigated using an in vitro model.

Because LN is caused by severe inflammation in the kidneys and glomerulus specifically, a model was designed to mimic this, using cytokines known to be significantly increased in patients with active LN compared with healthy controls and JSLE patients without renal disease. The cytokines were investigated individually to discern the effects of each specific inflammatory mediator and in a combination model to determine whether any additive or synergistic effects may be occurring.

Identification of novel urinary biomarkers has been of crucial importance in the study of LN, especially in children, as this will enable clinicians to actively manage the disease, decreasing the risk of damage accrual caused by disease flares over time (35). However, while this may slow the progression of disease, it will not prevent damage occurring. This can only be achieved by gaining a mechanistic understanding of the underlying processes. Previous work in our laboratories, and others, have identified a panel of novel urinary biomarkers specific to an active flare of LN (36, 39, 41, 42). Identifying the site of production of these biomarkers may identify novel potential therapeutic targets that could be used to prevent damage from occurring. Of note, however, this study demonstrated that podocytes themselves are unable to produce AGP or Tf under any of the conditions tested. Furthermore, the other five urinary biomarkers tested were expressed at relatively low levels at both the mRNA and protein level, and of these only VCAM-1 levels were statistically significantly affected by treatment. This indicates that podocytes are not the main source of the urinary biomarkers seen in LN but that these must be coming from another cell type or types within the kidney, from infiltrating immune cells recruited to the kidney in response to damage, or filtered through from the systemic circulation.

These data do not negate the role these biomarkers may be having in causing or contributing toward podocyte damage. This continues to be addressed in ongoing studies to further determine the damage mechanisms that may be occurring.

This study then aimed to determine the contribution of podocytes to the inflammatory environment observed in LN by using the in vitro model, since chronic inflammation is known to be a main driver of LN damage. Podocytes produce TNF-α, IL-6, IL-8, VEGF, GM-CSF, and M-CSF at relatively low levels under basal conditions. These cytokines must, therefore, be important for maintaining homeostasis in the glomerulus. The present study demonstrated that in response to inflammatory stimulation, there is a significant increase in the levels of IL-6, IL-8, VEGF, and M-CSF, as well as new production of IP-10 and IL-10.

Increased levels of IL-6 occur in LN patients with WHO class IV diagnoses compared with other subclasses of LN and healthy controls (22). IL-6 blockade may improve renal disease (23) and delay LN onset in experimental models (10). Tocilizumab (a fully humanized monoclonal antibody against the IL-6 receptor) has shown promise in phase 1 clinical trials with a reduction in active urinary sediment and autoantibody levels (21). IL-8 is important for neutrophil recruitment during an inflammatory response and is upregulated compared with healthy controls in SLE patients (32). The increased production from podocytes suggests that these cells may be involved in perpetuating the inflammatory response by recruiting neutrophils to the glomerulus.

VEGF is produced by podocytes under basal conditions where it maintains glomerular endothelial cell and mesangial cell survival (7). In our model an increase in the expression of VEGF is seen in response to damage. This mimics what is seen in an animal model of VEGF-A overexpression in which glomerular disease was induced (38) and an in vitro model of insulin sensitivity where increased VEGF was seen in response to insulin treatment (13). This suggests that a balance is required in VEGF levels where both too little and too much may be damaging within the glomerulus. M-CSF is important for the maturation of monocytes into macrophages and thus is involved in the damage caused by these cells within the glomerulus. Biomarker research has identified urinary levels of M-CSF as potential predictors for a flare of LN (37). IP-10 is a chemokine for monocyte/macrophages and T cells and thus is involved in the perpetuation of the inflammatory response. It has been shown to be increased in urine and serum of LN patients during active disease (1). IL-10 is involved in the pathogenesis of LN as it promotes the activation and differentiation of B cells, and levels are increased in the sera of patients that correlate with serological disease activity (19). B-N10, a murine antibody to IL-10, was used in a study in patients with relatively mild disease (25). These patients showed initial improvement but developed antibodies against the murine monoclonal antibody over time.

Having determined that podocytes can contribute significantly to the inflammatory environment by producing chemokines to recruit immune cells and cytokines that promote the differentiation and maturation of these immune cells, it was important to also determine how podocytes respond to the inflammatory environment. This was especially pertinent as the structural roles of podocytes have long been thought to be more important than their specific immune functions.

Podocytes are terminally differentiated cells and thus have a limited capacity for replenishment following damage-induced loss (2, 4). Foot process effacement, a loss of the usual interdigitating pattern of foot processes from neighboring cells, is a mechanism by which the amount of damage caused is reduced (24). Early damage is reversible, while sustained chronic damage cannot be rectified (12). This study demonstrated that in response to a single injurious stimulus, podocytes increase their intracellular calcium level leading to a reorganization of the actin cytoskeleton within 1 h. This caused a significant reduction in the area covered by cells in a monolayer and a loss of the slit diaphragms connecting the cells. However, after 24 h the healthy phenotype has been restored, and cells are once again in a continuous monolayer with interconnecting slit diaphragms. These data support what is seen in in vivo models of podocyte damage, in which acute damage is restored following removal of the injurious stimuli, while chronic damage results in loss of the podocytes and permanent damage (43).

These data demonstrated what occurs in our in vitro cytokine-based model. However, to develop a more physiologically relevant model, podocytes were incubated with sera from patients with LN. No modulation of the actin cytoskeleton was seen in this model. However, patients within the JSLE cohort study are routinely treated with steroids and immunosuppressant agents that may be reducing any effects that may otherwise be seen.

While this study demonstrated that the basal structure of the podocyte is restored 24 h after the initial damage, it is important to note that a reduction in the proinflammatory cytokine damage does not occur in human LN disease, and thus resolution is not reached. Future work will address the changes that occur in response to chronic stimulation to closer mimic human disease. This work was limited by being an in vitro model using cytokine treatments to mimic the pathogenesis of LN. However, it was important for gaining a mechanistic understanding of how damage is occurring in podocytes that can be expanded for future studies.

Taken together these data demonstrated that podocytes may represent an important therapeutic target for kidney-specific disease. Reducing the levels of specific proinflammatory cytokines within the kidney may attenuate both the exacerbation caused by the podocytes producing inflammatory mediators and specific damage to the podocytes themselves by these inflammatory mediators.

### 

#### Conclusion.

In response to our cytokine-induced in vitro model of LN, we demonstrated that podocytes produce proinflammatory cytokines and chemokines that are involved in the recruitment, maturation, and activation of immune cells and may, therefore, contribute to perpetuation of the inflammatory response. Podocytes respond in this model with foot process effacement that is preceded by an increase in intracellular calcium but not associated with increased cell death. The foot process structure is restored by 24 h, confirming that this is a temporary process in response to a single stimulus.

## GRANTS

This work was supported by the Alder Hey Children’s Kidney Fund, the UK’s Experimental Arthritis Treatment Centre for Children (supported by Arthritis Research UK, Alder Children’s NHS Foundation Trust, the Alder Hey Charity, and the University of Liverpool), and the National Institute of Health Research (NIHR) Alder Hey Clinical Research Facility for Experimental Medicine.

## DISCLOSURES

No conflicts of interest, financial or otherwise, are declared by the authors.

## AUTHOR CONTRIBUTIONS

R.D.W. and M.W.B. conception and design of research; R.D.W. performed experiments; R.D.W. analyzed data; R.D.W. interpreted results of experiments; R.D.W. prepared figures; R.D.W. drafted manuscript; R.D.W. and M.W.B. edited and revised manuscript; R.D.W. and M.W.B. approved final version of manuscript.
